# Exploring genomic variation associated with drought stress in *Picea mariana* populations

**DOI:** 10.1002/ece3.6614

**Published:** 2020-08-04

**Authors:** Joseph D. Napier, Guillaume de Lafontaine, Feng Sheng Hu

**Affiliations:** ^1^ Department of Plant Biology University of Illinois Urbana IL USA; ^2^ Department of Integrative Biology The University of Texas at Austin Austin TX USA; ^3^ Canada Research Chair in Integrative Biology of Northern Flora Université du Québec à Rimouski Rimouski QC Canada; ^4^ Department of Geology University of Illinois Urbana IL USA; ^5^ Department of Biology Washington University St. Louis MO USA; ^6^ Department of Earth and Planetary Sciences Washington University St. Louis MO USA

**Keywords:** Alaska, drought, genotype–environment associations, local adaptation, *Picea mariana*

## Abstract

Predicted increases in drought and heat stress will likely induce shifts in species bioclimatic envelopes. Genetic variants adapted to water limitation may prove pivotal for species response under scenarios of increasing drought. In this study, we aimed to explore this hypothesis by investigating genetic variation in 16 populations of black spruce (*Picea mariana*) in relation to climate variables in Alaska. A total of 520 single nucleotide polymorphisms (SNPs) were genotyped for 158 trees sampled from areas of contrasting climate regimes. We used multivariate and univariate genotype‐by‐environment approaches along with available gene annotations to investigate the relationship between climate and genetic variation among sampled populations. Nine SNPs were identified as having a significant association with climate, of which five were related to drought stress response. Outlier SNPs with respect to the overall environment were significantly overrepresented for several biological functions relevant for coping with variable hydric regimes, including osmotic stress response. This genomic imprint is consistent with local adaptation of black spruce to drought stress. These results suggest that natural selection acting on standing variation prompts local adaptation in forest stands facing water limitation. Improved understanding of possible adaptive responses could inform our projections about future forest dynamics and help prioritize populations that harbor valuable genetic diversity for conservation.

## Graphical abstract

Genetic variants adapted to water limitation may prove pivotal for species response under scenarios of increasing drought. In this study, we aimed to explore this hypothesis by investigating genetic variation in Alaskan populations of black spruce in relation to local climate. Our results suggest that natural selection acting on standing variation prompts local adaptation in forest stands facing water limitation.

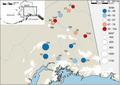

## 1 INTRODUCTION

Impending climate change is predicted to induce drastic shifts in the bioclimatic envelopes of forest species on most continents (Hughes, Cawsey, & Westoby, 1996; McKenney, Pedlar, Lawrence, Campbell, & Hutchinson, 2007; McKenney, Pedlar, Rood, & Price, 2011). These predictions suggest that many forest taxa will face challenges related to spatial redistribution and increased mortality risk due to drought and heat stress (Allen et al., 2010; Peng et al., 2011). In fact, latitudinal and altitudinal migrations have already been observed for many forest taxa (Lenoir, Gegout, Marquet, de Ruffray, & Brisse, 2008), and the negative impact of drought stress has been widely documented in tree species across the globe (Calder & Kirkpatrick, 2008; Chen & Luo, 2015; Ma et al., 2012; Peng et al., 2011). Prognostic climate scenarios suggest increased frequencies of summer drought in many regions, which may adversely impact trees that are particularly vulnerable to high conductivity losses following severe summer droughts (McDowell et al., 2008; Rosner et al., 2018; Zwieniecki & Secchi, 2015).

In response to predicted climate shifts, trees may exhibit one of several responses including range shifts, local extinction, or adaptation (Aitken, Yeaman, Holliday, Wang, & Curtis‐McLane, 2008; Christmas, Breed, & Lowe, 2016; de Lafontaine, Napier, Petit, & Hu, 2018). Because of the long generation time of many tree taxa, adaptations arising from new mutations may not be adequate to curb the effects of accelerating anthropogenic climate change (Allen et al., 2010; Huntley, 2007). Despite these concerns, locally adapted genetic variants are prevalent in many species (e.g., Alberto et al., 2013; Fournier‐Level et al., 2011; Riihimäki, Podolsky, Kuittinen, Koelewijn, & Savolainen, 2005; Santamaría et al., 2003; Savolainen, Pyhäjärvi, & Knürr, 2007). Adaptive responses from standing genetic variation in response to rapidly changing environments is a much faster process than mutations (Barrett & Schluter, 2008; de Lafontaine et al., 2018). Thus, local populations of widespread tree taxa that experience relatively warm and dry summer conditions in some portions of the species range may already contain in situ genotypes that are better adapted to drought stress relative to variants present in other regions (Du, Wang, Wang, Ueno, & de Lafontaine, 2020). Such molecular variants represent useful sources of genetic variation for managing populations to enhance resiliency to future climate shifts (Christmas et al., 2016) and could be potential targets for assisted gene flow to mitigate maladapted populations (Aitken & Whitlock, 2013).

The boreal forests of Alaska span a broad range of temperature and moisture gradients (Chapin et al., 2006; Shulski & Wendle, 2007). Conifer trees are common throughout this vast region, with black spruce (*Picea mariana*) being a dominant species under a variety of environmental conditions (Fryer, 2014). For instance, black spruce communities are exposed to warmer, drier summers in interior Alaska than more coastal regions, inducing episodes of stressful drought (Barber, Juday, & Finney, 2000; Shulski & Wendle, 2007). Decreased tree growth rates have been attributed to rising summer temperatures, and carbon isotope analyses have revealed pervasive drought stress of black spruce forests throughout interior Alaska (Walker, Mack, & Johnstone, 2015; Wolken et al., 2016). Dissimilar environmental regimes thus represent contrasting selective pressures within the geographic range of the Alaskan black spruce that could result in genetic divergence and differential local adaptations of populations. Although black spruce itself may not currently be under grave extinction threat from anthropogenic climate change due to its broad distribution, this system can provide valuable insights for other temperate–boreal conifer taxa that will be greatly impacted by future drought stress (Broadmeadow & Ray, 2005; Williams et al., 2012).

Genotype–environment associations (GEAs) are increasingly being used to assess genomic imprints consistent with natural selection, providing insights on adaptive responses of species to climate change (Hoban et al., 2016; Rellstab, Gugerli, Eckert, Hancock, & Holderegger, 2015; Sork et al., 2013). Many adaptive processes are expected to result in weak, multilocus signatures due to polygenic adaptation from standing variation, rather than selection on new mutations rapidly rising to fixation (Lind, Menon, Bolte, Faske, & Eckert, 2018; Savolainen, Lascoux, & Merilä, 2013). Detecting these weak signals through univariate GEAs is complicated by correcting for multiple comparisons (François, Martins, Caye, & Schoville, 2016). However, combining conventional univariate approaches with new multivariate techniques could retain the strengths of the univariate approach in detecting associations from genome scans while minimizing false positives without relying on stringent corrections that preclude the detection of weaker genomic signals (Forester, Lasky, Wagner, & Urban, 2018). In this study, we used a combination of multivariate and univariate GEA approaches to identify genetic variants statistically associated with key climate variables within a panel of single nucleotide polymorphisms (SNPs) located in expressed genes of the conifer *Picea glauca* (Pavy et al., 2013). We hypothesized that the standing genetic variation in black spruce from contrasting hydric regimes would display genomic imprints consistent with local adaptation to varying drought conditions.

## 2 MATERIALS AND METHODS

### 2.1 DNA extraction and genotyping assay

**FIGURE 1:**
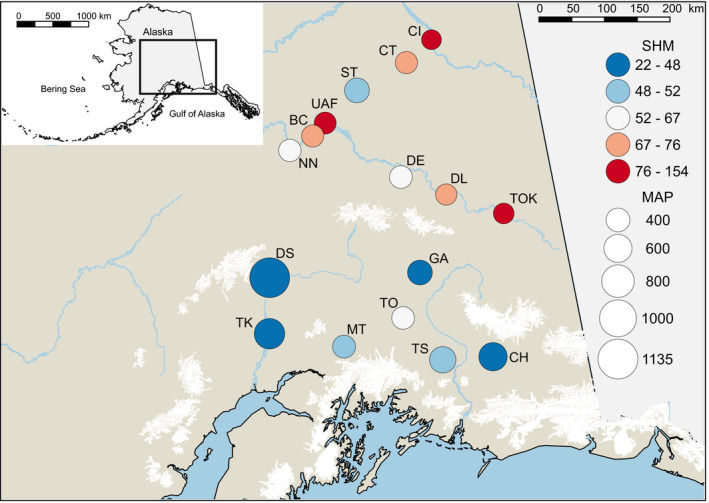
Location of the 16 black spruce populations sampled in Alaska. Circle color indicates the summer heat‐to‐moisture index (SHM) with lower values indicating a cooler, wetter summer and higher values indicating a warmer, drier summer environment. Circle size proportional to mean annual precipitation (MAP)

We sampled 158 black spruce individuals from 16 sites in Alaska with a wide range (ca. sevenfold difference) of summer heat‐to‐moisture index (SHM), where lower SHM values indicate cooler, wetter conditions and higher values indicate hotter, drier summer conditions (Figure 1). At each site, needles from ca. 10 individuals spaced > 100 m apart were sampled and dried in silica gel. For each individual, DNA was extracted from 18 mg of homogenized dry tissue using Nucleospin 96 Plant II (Macherey‐Nagel Inc.). The SNP genotyping assay was performed using an Illumina iSelect Infinium array (Illumina Inc.) developed for population genetic analysis, genomic prediction, and linkage mapping in *Picea glauca* (Pavy et al., 2013). Specifically, the array is a subset of the PgLM3 genotyping SNP (Pavy et al., 2013) consisting of 6,000 beads with one bead per SNP, and each of these SNPs was related to a different gene designed to maximize the number of uniquely informative SNPs and gene loci assayed in our panel. A total of 5,308 SNPs were successfully genotyped. From these, 4,788 SNPs were discarded based on a call rate cutoff of 0.75 (average call rate ~ 0.97; median call rate ~ 0.99) and a minor allele frequency (MAF) of 0.05. The largest number of removed loci was either monomorphic (*n* = 1,790) or uninformative given their low MAF (*n* = 2,902) in the sampled Alaskan black spruce populations. Of the remaining SNPs, 96 were removed based on the low call rate, resulting in a final dataset of 520 polymorphic SNPs. According to Pavy et al. (2013), interspecies SNP transferability of the PgLM3 genotyping array to black spruce is 18%. Our selection criteria for SNP inclusion are more stringent compared with those of Pavy et al. (2013) (call rate = 0.5; MAF = 0.01), which explains the lower SNP transferability (9%) in our dataset relative to theirs.

### 2.2 Neutral genetic structure

As with any other population genomic approach, GEAs may be biased by the presence of genetic structure across sampled populations if this structure is not accounted for (Excoffier, Hofer, & Foll, 2009; de Villemereuil, Frichot, Bazin, Francois, & Gaggiotti, 2014). We evaluated neutral genetic structure using the Bayesian clustering algorithm implemented in STRUCTURE v2.3.4 (Falush, Stephens, & Pritchard, 2003; Pritchard, Stephens, & Donnelly, 2000). In STRUCTURE, an admixture model with correlated allele frequencies was used with no prior information. The analysis consisted of 20,000 burn‐in steps and 100,000 replicates of 1–10 genotypic groups (*K*), each of which was run 10 times. The optimal *K* value was evaluated based on ∆*K* implemented in STRUCTURE HARVESTER (Earl & vonHoldt, 2012; Evanno, Regnaut, & Goudet, 2005) and a visual comparison of results from each *K*. This analysis, including all 520 SNPs, is assumed to be largely representative of neutral variation. However, this assumption could be violated because the SNP array was specifically designed for the inclusion of functional genes potentially submitted to natural selection. Hence, we tested each SNP for departures from Hardy–Weinberg equilibrium (HWE) using the *pegas* package in R version 3.5.1 (Paradis, 2010; R Core Team, 2018) and then reran STRUCTURE using only the putatively neutral SNPs that did not violate the assumptions of HWE. Because the two resulting matrices of ancestry (i.e., two sets of STRUCTURE *Q*‐values) were highly correlated (see results), subsequent analyses relied on the initial STRUCTURE analysis (i.e., including all 520 SNPs) to account for the neutral genetic structure of the sampled black spruce populations (Note S1).

### 2.3 Genotype–environment association and outlier analyses

Among GEA approaches, univariate methods, which test one locus and one predictor variable at a time, are the most commonly used (Forester et al., 2018; Rellstab et al., 2015). These approaches perform well for detecting significant associations but may be prone to false positives without corrections or may be overly conservative when correcting for the sheer number of tests associated with genomic studies (François et al., 2016). Consequently, univariate GEA methods struggle to detect loci under weak selection (Forester et al., 2018). Multivariate approaches are more effective to uncover weak, multilocus genomic imprints consistent with soft sweeps on standing genetic variation as they consider how groups of markers covary with a set of environmental predictors (as reviewed by Le Corre & Kremer, 2012; Rellstab et al., 2015). However, because the multivariate approach looks for loci that exhibit unusual patterns across all variables, a locus may appear as an outlier even when it is not strongly related to any given climate variable.

We thus relied on overlapping results of GEA from multivariate and univariate analyses to detect statistically significant correlations between genotypes and climatic variables. These analyses were used jointly to identify a set of SNPs displaying associations between allele frequencies and environmental gradients, based on a series of climatic variables extracted for each sample location using from the Climate WNA database, which generates scale‐free climate data through a combination of bilinear interpolation and elevation adjustments (Dormann et al., 2013; Wang, Hamann, Spittlehouse, & Carroll, 2016). A total of eight environmental variables were selected (mean annual temperature, mean warmest month temperature, mean coldest month temperature, continentality, mean annual precipitation, annual heat‐to‐moisture index, summer heat‐to‐moisture index, and summer solar radiation) after screening the full set of annual and seasonal climate variables for collinearity (*r* < .80) from the baseline Climate WNA dataset (Dormann et al., 2013; Wang et al., 2016).

We used redundancy analysis (RDA) to identify SNP loci associated with the environmental variables in our final dataset as the multivariate analysis for detecting probable adaptive variants in this study (Forester et al., 2018). RDA is a constrained ordination technique that can detect putative selective processes that create even weak molecular signatures by determining how a set of loci covary with the environment (Forester et al., 2018; Rellstab et al., 2015). According to Forester et al. (2018), RDA consistently demonstrated a desirable combination of high true positives and low false positives across all levels of selection. One caveat to this approach is that neutral population genetic structure may bias the results leading to false positives (Excoffier et al., 2009). To account for this possibility and the effect of geographic distance, we slightly modified the approach to a partial redundancy analysis (pRDA) by adding a conditioned matrix consisting of geographic coordinates (latitude and longitude) and ancestry coefficients (*Q*‐values) from STRUCTURE analysis. Using the R package *vegan*, we conducted the pRDA (Oksanen et al., 2016). First, multivariate linear regression was used on the minor allele counts (i.e., 0, 1, or 2) of each locus and the extracted climate variables to produce a matrix of fitted values. Principal components analysis (PCA) was then performed on this matrix to generate canonical axes that were linear combinations of the predictors (Legendre & Legendre, 2012). For each significant RDA axis, we extracted the loadings, which were approximately normally distributed. SNPs loading at the tails are more likely under selection by the predictors (i.e., climate variables), so we identified all markers that fell within 2.5 standard deviations (two‐tail *p*‐value = .012) from the center as putative loci under selection (Forester, Jones, Joost, Landguth, & Lasky, 2016; Forester et al., 2018).

We used hierarchical Bayesian mixed modeling as the univariate approach in this study. Specifically, latent factor mixed models (LFMMs) were used to identify black spruce allele–environment correlations with neutral population structure modeled as "latent factors" (Frichot, Schoville, Bouchard, & Francois, 2013). The LFMM approach was implemented in the R package LEA (Frichot, François, & O'Meara, 2015). The number of latent factors (*K*) was determined by comparing results from the STRUCTURE analysis (i.e., delta *K*) with the spare non‐negative matrix factorization (smnf) function available in LEA that estimates ancestry coefficients with smnf (i.e., cross‐entropy criterion for each *K*). A model was run for each of the eight environmental variables. Loci statistically associated with a climate variable were then compared with the results from our partial RDA. Those SNPs jointly flagged by both approaches were assumed as candidate loci, putatively under selection by climate (Rellstab et al., 2015).

GEAs represent one of the two key approaches for identifying the signatures of selection and related candidate SNPs. The other approach identifies loci with extreme allele frequency differences among populations relative to overall population genetic structure, with such patterns potentially indicating regions under selection (Hoban et al., 2016; Rellstab et al., 2015). These differentiation‐based outlier approaches often rely on population genetic models that make sweeping assumptions that are often problematic for empirical studies (detailed in Flanagan, Forester, Latch, Aitken, & Hoban, 2018). Accordingly, we utilized a method that does not rely on underlying population genetic models, pcadat, to further validate potential candidate SNPs (Luu, Bazin, & Blum, 2017). Specifically, pcadapt was used to generate principal components that estimated population structure and identify candidate markers with respect to how they are related to this structure. These results were compared with those from the two GEA approaches to provide further evidence of potential selection in sampled Alaskan black spruce populations.

Lastly, we used available Gene Ontology (GO) annotations to explore possible functional implications of the genes associated with climate across the sampled black spruce populations. TAIR (The Arabidopsis Information Resource; Lamesch et al., 2012) annotations of the candidate loci were retrieved based on their homology with sequences from the most complete gene catalogue of *Picea glauca*, the closest relative of *Picea mariana* with available annotations (Rigault et al., 2011). To determine whether the genomic imprints may be related to environmental factors, statistical overrepresentation tests were performed using the PANTHER classification system (Mi et al., 2017; Mi, Muruganujan, Casagrande, & Thomas, 2013). This analysis determines whether the outlier SNPs identified by the partial RDA approach were overrepresented for biological functions (Mi et al., 2013) with respect to the list of 520 SNPs assayed. The enrichment analysis was run on all SNPs identified by partial RDA, rather than the few markers jointly identified by both approaches, in order to achieve sufficient statistical power and include more genes putatively under weak selection. The presence of significant enrichment was determined by using Fisher's exact tests of biological processes related to Gene Ontology (*α* = .05). We acknowledge that our approach to annotating adaptive SNPs and making additional functional interpretations assumes that similarity between *P. mariana* and *P. glauca* genomes, and similarity between predicted *P. glauca* Gene Ontology (GO) terms and *Arabidopsis thaliana* TAIR orthologues, is sufficient to reflect possible gene functions in *P. mariana*. As a result, we cautiously interpret these results in view of these limiting assumptions.

## 3 RESULTS

STRUCTURE analysis of the 520 polymorphic SNPs in our final dataset supported two distinct genetic clusters (*K* = 2; Figure S1). Assessing all 520 SNPs for HWE using *pegas* and correcting for multiple testing procedures (Bonferroni's correction at *α* = .05) indicated that 201 SNPs did not violate the assumptions of HWE. Running STRUCTURE using this subset yielded nearly identical results; the matrices of ancestry coefficients (*Q*‐values) from the two STRUCTURE analyses were strongly correlated (Pearson's *r* = .94). We used the *Q*‐values from the analysis of all SNPs to remove the effects of neutral genetic structure in the pRDA.

**FIGURE 2:**
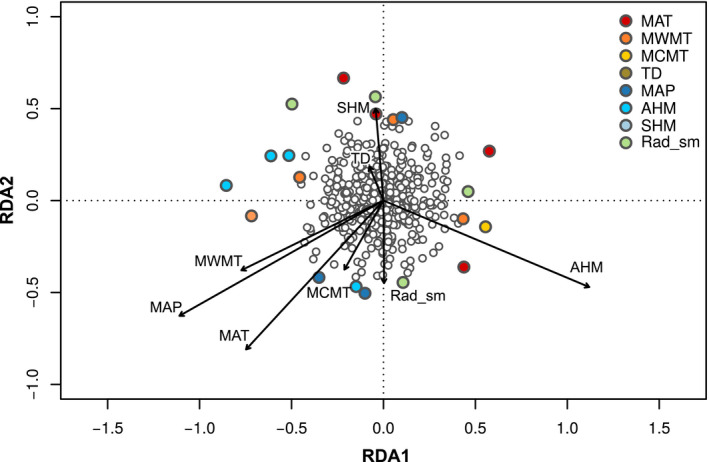
Results from the partial redundancy analysis relating genetic variability in Alaskan black spruce with downscaled climate variables. All 520 SNPs are plotted by their score on the first and second significant axes (RDA 1, *x*‐axis and RDA 2, *y*‐axis, respectively). Significant outlier SNPs (*n* = 20) are colored according to their most correlated climate variable: mean annual temperature (MAT), mean warmest month temperature (MWMT), mean coldest month temperature (MCMT), continentality (TD), mean annual precipitation (MAP), annual heat‐to‐moisture index (AHM), summer heat‐to‐moisture index (SHM), and summer solar radiation (Rad_sm)

The multivariate pRDA conditioned on geographic location and neutral genetic structure resulted in two significant canonical axes according to *F* tests from an analysis of variance (*p* < .05; Table S2). Both axes followed an approximately normal distribution, which allowed us to identify outlier loci based on standard deviations (Figure S2). We uncovered 11 and 10 outlier SNPs on the first and second canonical axis, respectively. One SNP was flagged as an outlier on both axes, leaving a total of 20 unique outlier SNPs (Figure 2).

**FIGURE 3:**
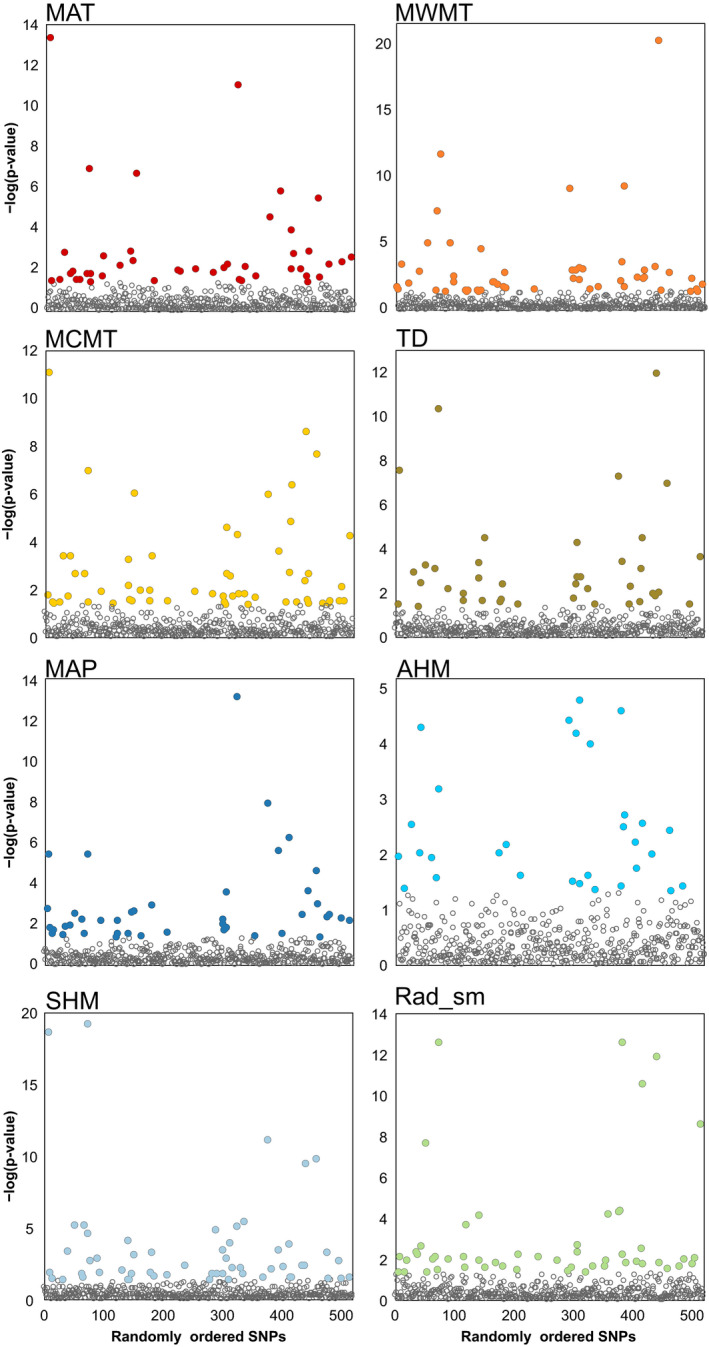
Manhattan plots with colored circles depicting SNPs significantly associated with a climatic variable according to latent factor mixed modeling (*K* = 2 latent factors for each model). See Figure 2 legend for full terms of abbreviated climate variables

The univariate LFMM analysis revealed that 30–58 SNPs were statistically associated with each of the eight environmental–climatic variables included in our study (Figure 3 and Table S3). Nine of the 20 outlier SNPs flagged by partial RDA were also identified in one of the LFMMs (Table 1 and Figure S3). These nine SNPs, at the intersection of results from our multivariate and univariate statistical approaches to GEA, were assumed to be the most likely candidate loci of potential adaptation to selection by climate and were deemed putative "adaptive" genetic variants (Rellstab et al., 2015).

**TABLE 1:** List of the nine candidate genes encompassing the SNP loci jointly identified by the two genotype–environment associations (GEAs)

Candidate gene accession in GCAT gene catalogue (Rigault et al., 2011)	Outlier on pRDA axis	Climate variable with strongest association in pRDA	LFMMs in which associated SNP was significant	TAIR description	Biological function	Known response to abiotic stress	Outlier with respect to neutral genetic structure in pcadapt
GQ03105_E17	1	MAT	MAT, MCMT, MAP	Glutathione peroxidase 6	Response to cadmium ion, response to salt stress	Drought response: cadmium stress signaling controls the expression of genes in drought stress signal pathways (Oono et al., 2014)	Yes
GQ03515_C04	1	Rad_sm	MWMT, MAP, AHM, SHM	Protein‐related	Senescence/dehydration‐associated protein‐like protein	Drought response	Yes
GQ03615_N10	1	MAT	MAP	Dehydratase family	Encodes enzymes involved in BCCA biosynthesis	Drought response: BCAAs accumulate in response to drought (Huang & Jander, 2017)	No
GQ03514_F14	2	MAT	MAT, MCMT, MAP	Cupin superfamily protein	Overexpression results in drought resistance	Drought response: Overexpression leads to the salt and drought stress resistance (Rezapoor, Aghdasi, & Sadeghipoor, 2017)	No
GQ03712_C02	2	MAP	MAP	Cinnamate‐4‐hydroxylase	Mutations in this gene impact phenylpropanoid metabolism and growth	Drought response: Phenylpropanoid metabolism is stimulated by abiotic stresses such as drought (Cabane et al., 2012)	Yes
GQ0259_L19	1	AHM	AHM	Unavailable	Unknown		No
GQ04103_E16	1	MWMT	MWMT, SHM, Radsm	Diacylglycerol kinase 5	Unknown		No
GQ04102_G23	1	Rad_sm	MAT, MWMT, MCMT, TD, SHM, Rad_sm	Methyl esterase 1	Encodes protein involved in hydrolysis		No
GQ0163_M01	2	MAP	MAP	Protein‐related	Involved in hydrolase activity and biological processes		No

Note

For each SNP, candidate gene accession in GCAT white spruce gene catalogue (Rigault et al., 2011), TAIR description, outlier status with regard to neutral genetic population structure, and biological function are listed along with results from other studies detailing their role in abiotic response to stress.

Abbreviations: AHM, annual heat‐to‐moisture index; MAP, mean annual precipitation; MAT, mean annual temperature; MCMT, mean coldest month temperature; MWMT, mean warmest month temperature; Rad_sm, summer solar radiation; SHM, summer heat‐to‐moisture index; TD, continentality.

Results from pcadapt revealed 46 SNPs were statistical outliers with respect to the neutral genetic structure represented by five retained principal components. Three of the nine outlier SNPs flagged by both the partial RDA and LFMMs were also identified in this approach (Table 1). Steady outlier detection across environmental gradients and with respect to neutral genetic structure is consistent with a signature of natural selection across Alaskan black spruce. Hence, the three SNPs identified across all methods represent the most robust candidates as being under selection.

**FIGURE 4:**
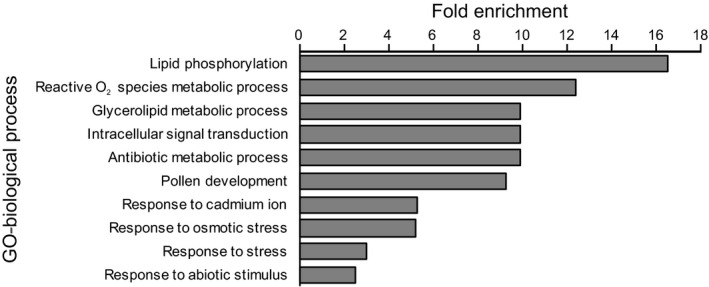
Fold enrichments for all the biological processes that were found to be statistically significant in a gene enrichment analysis. All outliers from the partial redundancy analysis were compared against the background reference of all 520 SNPs. All significant biological processes detected were overrepresented at a rate of two and half more times likely than would be randomly expected when compared to all background SNPs (i.e., at least a fold enrichment of 2.5)

The genes encompassing the nine candidate SNPs from GEAs were annotated based on their homology with sequences from the most complete gene catalogue of *Picea glauca* (Rigault et al., 2011) to assess their potential biological functions (Table 1). Of the nine SNPs, five (GQ03105_E17, GQ03515_C04, GQ03615_N10, GQ03514_F14, and GQ03712_C02) are reportedly related to drought responses, two (GQ04102_G23 and GQ0163_M01) are related to hydrolysis, and the remaining two (GQ0259_L19 and GQ04103_E16) are not currently annotated (Table 1). Based on Gene Ontology annotations from TAIR, enrichment tests using the PANTHER classification system (Mi et al., 2013) showed that the 20 gene SNPs flagged by pRDA have biological functions that distinguish them from the full set of 520 SNPs assayed in the study. These gene SNPs are significantly overrepresented for several biological functions relevant for coping with variable hydric regime, including response to abiotic stimulus, stress response, pollen development, and osmotic stress response (Figure 4 and Table S4).

## 4 DISCUSSION

With mounting evidence that black spruce populations in interior Alaska experience drought stress (Walker et al., 2015; Wolken et al., 2016), we explored the possibility that the standing genetic variation of this species displays a genomic imprint consistent with local adaptation to varying hydric regimes. Black spruce grows under warmer and drier conditions in the Alaskan interior than in the more southern sites closer to the northern Pacific coast (Chapin et al., 2006; Shulski & Wendle, 2007; Wang et al., 2016). Our sampled populations span these climatic differences (Figure 1). Joint inferences from a pRDA and LFMMs identified nine SNPs that displayed significant associations with climate variables, suggesting potential local adaptation to environmental selective pressures (Rellstab et al., 2015; Sork et al., 2013).

Functional annotations of the candidate adaptive genetic variants identified in our study were based on a genome from the closest *Picea* relative available (*P. glauca*) and not the focal species, and we were mainly concerned with identifying environmental–climatic factors driving adaptive evolution in *P. mariana*. Therefore, we present a cautious interpretation of our functional results, considering these to represent preliminary findings until a full *P. mariana* reference genome becomes available. Gene annotations of the nine candidate SNPs shared between results from multivariate and univariate GEA approaches revealed that five of these SNPs were related to drought stress response pathways (Lamesch et al., 2012; Table 1). Among the five candidate SNPs related to drought, three were also identified as outliers with respect to neutral genetic structure, making these the most likely candidates as putatively being under selection (Table 1). Moreover, gene enrichment analysis of the outliers from the partial RDA indicated that the osmotic stress response is statistically overrepresented in the outliers when compared to the background of all 520 SNPs (Figure 4 and Table S4). The five candidate loci suggest variable possible mechanisms of adaptation (Table 1). One of these loci is related to cinnamate‐4‐hydroxylase, which is a pivotal enzyme in drought response pathways (Table 1; Betz, McCollum, & Mayer, 2001; Cabane, Afif, & Hawkins, 2012). A previous study on loblolly pines (*Pinus taeda* L.) also found that genetic regions related to cinnamate‐4‐hydroxylase were under selection in response to drought (Koralewski, Brooks, & Krutovsky, 2014). Our analysis of GEAs also highlighted loci related to glutathione peroxidase (Table 1). An experimental study on the drought‐tolerant conifer Aleppo pine (*Pinus halepensis*) found that genetic regions related to glutathione peroxidase were upregulated in response to experimental drought (Fox et al., 2018). Future studies exploring these genetic regions and ascertainment of candidate SNPs through inclusion of phenotypes (i.e., reciprocal transplant experiments using common gardens, and controlled experiments on fitness in varying drought conditions) will be necessary to validate these putatively adaptive genetic variants and to understand in greater detail how black spruce populations in Alaska and other regions adapt to drought conditions.

Our STRUCTURE analyses revealed two distinct lineages that were spatially clustered across the sampled black spruce populations (Figure S4). Although this analysis was primarily used to control neutral genetic structure in this study, the two clusters probably reflect distinct black spruce glacial lineages previously isolated in refugia in Alaska or other areas of North America during the Last Glacial Maximum (LGM), and that subsequently recolonized Alaskan portions of the species range after the LGM (Gerardi, Jaramillo‐Correa, Beaulieu, & Bousquet, 2010). Since the uplift of the Alaska Range, the interior populations have likely been exposed to a greater selective pressure imposed by low effective moisture relative to the populations in the more southerly sites. We hypothesize that this selective pressure continued during the last glaciation and the early Holocene, when the region was much drier than during the late Holocene (Edwards, Mock, Finney, Barber, & Bartlein, 2001; Kaufman et al., 2016). Both limited gene flow across the Alaska Range and strong selection pressure impeding maladapted immigrants in these contrasted environments have likely contributed to the maintenance of neutral genetic divergence between the two regions (Lenormand, 2002; Nosil, Vines, & Funk, 2005; Wang & Bradburd, 2014). Thus, given the significant relationship between genetic variation and climate even when accounting for neutral genetic structure, the genomic imprints consistent with local adaptation reported in this study probably reflect long‐term adaptive response of black spruce to climate contrasts among our sites since the late Quaternary (late Pleistocene to Holocene). The degree to which adaptation signals in modern populations are due to the accumulation of past genetic variation in response to paleoenvironments is difficult to disentangle in natural settings (de Lafontaine et al., 2018). Although the role of adaptation to past environments has been acknowledged as a crucial mechanism in facilitating long‐term persistence (Davis & Shaw, 2001), evidence remains limited. Our results suggest that Alaskan spruce populations have been subjected to long‐term, contrasting selective pressures and thus represent an appropriate biogeographical model to explore the role of historical evolutionary processes in driving adaptive genetic variation among modern forest tree populations (de Lafontaine et al., 2018).

Forests are vulnerable to increased frequency and severity of droughts in the Anthropocene, and the level of this vulnerability is underestimated (Allen, Breshears, & McDowell, 2015). For instance, boreal “browning” (i.e., forests with reduced growth that turn brown due to increasingly warming summers) are not currently included in estimates of future vulnerability and persistence (Sitch et al., 2015). Given these concerns, it is more timely than ever that we understand the various possible responses of forest taxa to anthropogenic climate shifts and consider management steps that can be taken now to mitigate the negative effects of such shifts. Our results, along with those from several recent studies (Bansal, Harrington, Gould, & St Clair, 2015; Du et al., 2020; Liepe, 2014), suggest that natural selection acting on standing genetic variation prompts local adaptation to drought stress in forest stands facing drought conditions. Given projected severe droughts in many forested regions concomitant with ongoing and future projected climate change (Law, 2014), genetic diversity that has evolved through millennia of dramatic climatic fluctuation, such as the molecular variants identified in our study, is particularly valuable and should be prioritized for conservation (de Lafontaine et al., 2018; Mitrovski, Hoffmann, Heinze, & Weeks, 2008). Specifically, for managed conifer stocks facing impending drought stress (i.e., Broadmeadow & Ray, 2005), the genetic markers identified through GEAs represent promising avenues for marker‐assisted selection to mitigate the detrimental effects of drought (see Prunier, Laroche, Beaulieu, & Bousquet, 2011). Specifically, detected signals of adaptation to local climate conditions suggest assisted gene flow from historically drought‐stressed populations to maladapted tree populations could safeguard forest trees from future climate shifts (Aitken & Whitlock, 2013). Such conservation efforts may help ensure persistence and resilience of natural populations to future climate extremes (Reusch, Ehlers, Hammerli, & Worm, 2005; Sgro, Lowe, & Hoffmann, 2011).

## CONFLICT OF INTEREST

The authors have no competing interests to declare.

## AUTHOR CONTRIBUTIONS

**Joseph D. Napier:** Conceptualization (lead); data curation (lead); formal analysis (lead); methodology (lead); writing – original draft (lead); writing – review and editing (lead). **Guillaume de Lafontaine:** Conceptualization (supporting); data curation (supporting); formal analysis (supporting); methodology (supporting); writing – original draft (supporting); writing – review and editing (supporting). **Feng Sheng Hu:** Conceptualization (supporting); funding acquisition (lead); Resources (lead); writing – original draft (supporting); writing – review and editing (supporting).

## Supporting information

Appendix S1

## Data Availability

Genetic data are available on Dryad repository (https://doi.org/10.5061/dryad.tx95x69vn).
