# Supplementary material for: Exploring genomic variation associated with drought stress in Picea mariana populations
Source: Ecol Evol. 2020 Aug 4;10(17):9271–82. doi: 10.1002/ece3.6614 (PMC7487243; doi:10.1002/ece3.6614)
Supplement: Supplementary file 1 — Appendix S1 [file ECE3-10-9271-s001.docx]

**Appendix S1**

**Exploring genomic variation associated with drought stress in *Picea* *mariana* populations**

Joseph D. Napier, Guillaume de Lafontaine, Feng Sheng Hu

**Note S1. *Correctly accounting for the neutral genetic structure of the sampled black spruce populations***

The genotyping method for the sampled Alaskan black spruce populations was a SNP array where each SNP was putatively linked to a gene of interest. This inherently suggests there could be departures from Hardy–Weinberg equilibrium (HWE) as these genes might be under natural selection. As such, these SNPs could be biased for approximating the neutral genetic structure of the black spruce populations. Accordingly, each SNP was tested for departures from HWE using the hw.test function in the pegas package in R version 3.5.1 (Paradis, 2010; R Core Team, 2018). After accounting for multiple testing using a Bonferroni correction, we found that 201 SNPs did not violate the tenants of HWE. Using these putatively neutral SNPs we re-ran STRUCTURE. We then compared the matrices of ancestry (STRUCTURE *Q*-values) from each run (with all SNPs versus the subset that did not violate the assumptions of HWE) and found a strong correlation (Pearson’s *r* = 0.94) suggesting our initial STRUCTURE run adequately captured the neutral genetic structure of the Alaskan black spruce populations.

**TABLES AND FIGURES**

**Table S1**. Location of sampling locations of Alaskan black spruce populations and associated climatic conditions.

| Site | Longitude (°W) | Latitude (°N) | MAT | MWMT | MCMT | TD | MAP | AHM | SHM | Rad_sm |
| --- | --- | --- | --- | --- | --- | --- | --- | --- | --- | --- |
| TS | 145.12224 | 61.46149 | -3.4 | 11.7 | -19.8 | 31.5 | 526 | 12.5 | 49.2 | 17.4 |
| UAF | 147.86295 | 64.86517 | -2.5 | 16.1 | -21.0 | 37.1 | 317 | 23.8 | 79.6 | 15.9 |
| DE | 145.65791 | 64.01648 | -3.2 | 15.1 | -21.9 | 37 | 352 | 19.3 | 60.5 | 16.3 |
| BC | 148.3006 | 64.70383 | -2.7 | 16.1 | -21.8 | 37.9 | 328 | 22.2 | 75.7 | 16.1 |
| TO | 146.11227 | 62.09729 | -3.9 | 12.9 | -21.5 | 34.4 | 348 | 17.5 | 61.6 | 17.8 |
| NN | 149.06471 | 64.53096 | -3.4 | 15.7 | -21.7 | 37.4 | 338 | 19.7 | 67.3 | 16.3 |
| DS | 149.95377 | 62.81011 | -0.1 | 14.0 | -13.1 | 27.1 | 1135 | 8.7 | 22.1 | 19.3 |
| MT | 147.91644 | 61.79246 | -2.6 | 11.9 | -15.8 | 27.6 | 362 | 20.4 | 52.4 | 18.9 |
| TOK | 142.67598 | 63.31484 | -4.4 | 14.6 | -25.4 | 40.0 | 259 | 21.5 | 78.6 | 16.6 |
| ST | 146.72792 | 65.26804 | -3.1 | 14.3 | -20.0 | 34.3 | 449 | 15.4 | 48.7 | 16.4 |
| TK | 150.05964 | 62.04468 | -0.2 | 15.5 | -16.7 | 32.3 | 708 | 13.8 | 41.2 | 16.5 |
| DL | 144.33131 | 63.69484 | -4.3 | 14.6 | -27.2 | 41.7 | 289 | 19.9 | 71.2 | 17.0 |
| CH | 143.6778 | 61.40377 | -1.7 | 13.1 | -17.3 | 30.4 | 611 | 13.5 | 40.8 | 18.9 |
| GA | 145.4494 | 62.68601 | -4.0 | 12.6 | -20.8 | 33.4 | 446 | 13.5 | 48.0 | 16.9 |
| CT | 145.00226 | 65.56298 | -5.0 | 15.2 | -25.7 | 40.9 | 331 | 15.2 | 68.0 | 15.9 |
| CI | 144.06837 | 65.82568 | -6.5 | 17.1 | -27.7 | 44.8 | 220 | 15.7 | 153.9 | 16.2 |

MAT: Mean annual temperature

MWMT: Mean warmest month temperature

MCMT: Mean coldest month temperature

TD: Continentality

MAP: Mean annual precipitation

AHM: Annual heat-to-moisture index

SHM: Summer heat-to-moisture index

Rad_sm: Summer solar radiation

**Table S2**. Results of the analysis of variance used to determine the number of significant canonical axes for the partial redundancy analysis (permutations = 999). Two axes (shown in bold) were used to detect multivariate outlier loci.

|  | DF | Variance | *F* statistic | *p*-value |
| --- | --- | --- | --- | --- |
| **RDA1** | **1** | **1.635** | **1.5273** | **0.003** |
| **RDA2** | **1** | **1.45** | **1.3544** | **0.046** |
| RDA3 | 1 | 1.336 | 1.2472 | 0.28 |
| RDA4 | 1 | 1.253 | 1.1704 | 0.581 |
| RDA5 | 1 | 1.189 | 1.1103 | 0.738 |
| RDA6 | 1 | 1.121 | 1.0466 | 0.866 |
| RDA7 | 1 | 1.097 | 1.0245 | 0.741 |
| RDA8 | 1 | 0.962 | 0.8979 | 0.899 |
| Residual | 146 | 156.344 |  |  |

**Table S3.** The number of significant loci for each of the eight latent factor mixed models (LFMM). One univariate model with two latent factors was run for each of the eight climate variables: mean annual temperature (MAT), mean warmest month temperature (MWMT), mean coldest month temperature (MCMT), continentality (TD), mean annual precipitation (MAP), annual heat-to-moisture index (AHM), summer heat-to-moisture index (SHM), and summer solar radiation (Rad_sm).

|  | MAT | MWMT | MCMT | TD | MAP | AHM | SHM | Rad_sm |
| --- | --- | --- | --- | --- | --- | --- | --- | --- |
| Number of Significant Loci | 44 | 54 | 58 | 40 | 43 | 30 | 56 | 52 |

**Table S4.** Statistically significant results of the gene enrichment analysis for the biological processes associated with the outliers found in the partial redundancy analysis compared with the reference of all 520 SNPs analyzed for the black spruce populations. Enrichments for the listed biological processes were significant at an alpha threshold of 0.05.

| **GO biological process complete** | **Fold Enrichment** | ***p*-value** |
| --- | --- | --- |
| lipid phosphorylation | 16.52 | 0.013 |
| reactive O2 species metabolic process | 12.39 | 0.020 |
| glycerolipid metabolic process | 9.91 | 0.027 |
| intracellular signal transduction | 9.91 | 0.027 |
| antibiotic metabolic process | 9.91 | 0.027 |
| pollen development | 9.29 | 0.007 |
| response to cadmium ion | 5.31 | 0.024 |
| response to osmotic stress | 5.22 | 0.009 |
| response to stress | 3.05 | 0.001 |
| response to abiotic stimulus | 2.53 | 0.047 |


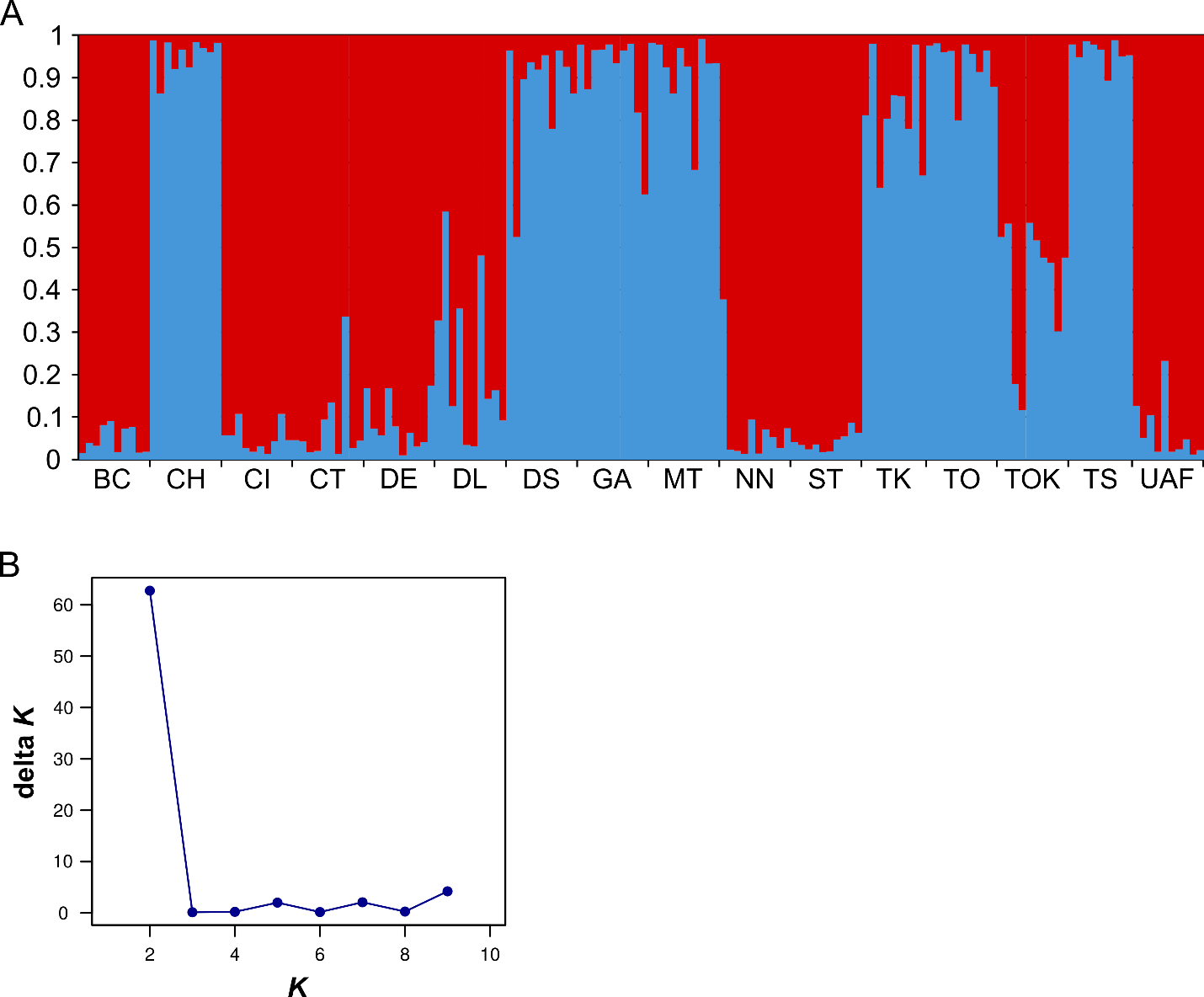


**Fig. S1.** Neutral genetic structure across sampled black spruce populations in Alaska. (A) Bar graphs represent the multilocus genetic assignment of each individual to a STRUCTURE group. Two distinct genetic groups were detected for sampled Alaskan black spruce populations by the clustering algorithm implemented in SRUCTURE (Pritchard et al., 2000; Falush et al., 2003). (B) The presence of two clusters was further supported as the most likely scenario by calculating ∆*K* in STRUCTURE HARVESTER (Evanno et al., 2005; Earl and vonHoldt, 2011).

**
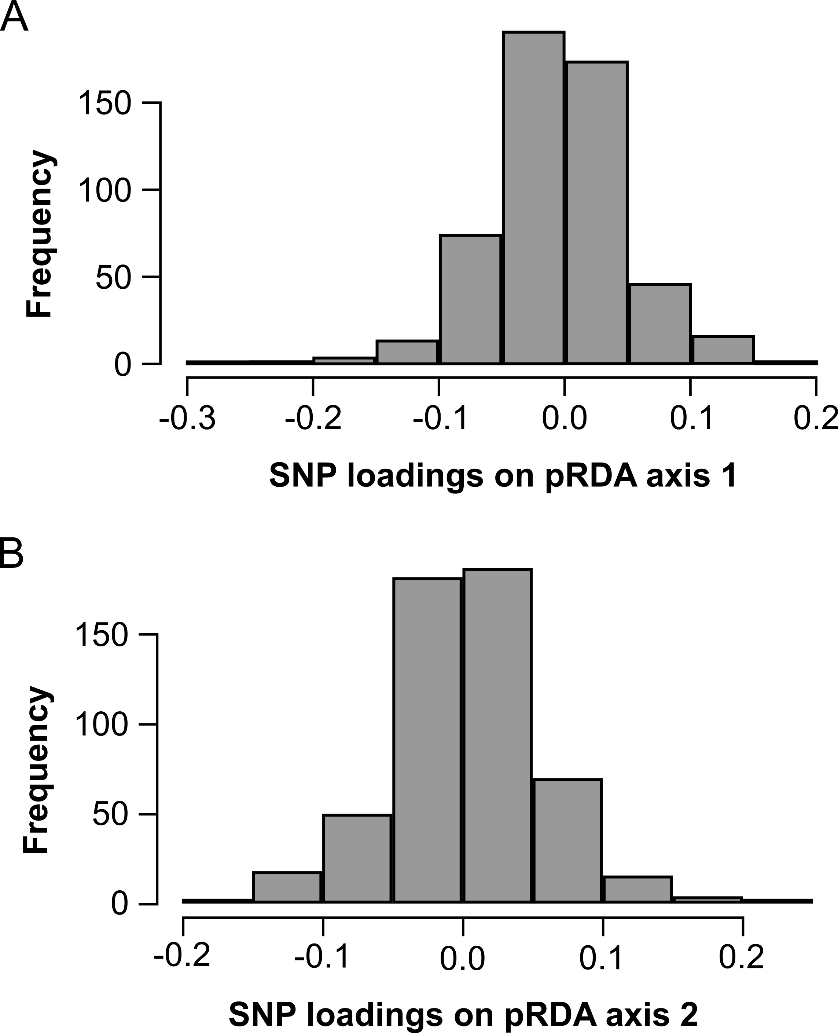
**

**Fig. S2.** Histograms of the distribution of SNP scores from the partial redundancy analysis on the first and second canonical axes. Both distributions are approximately bell-shaped and do not exhibit any significant deviations from normality.


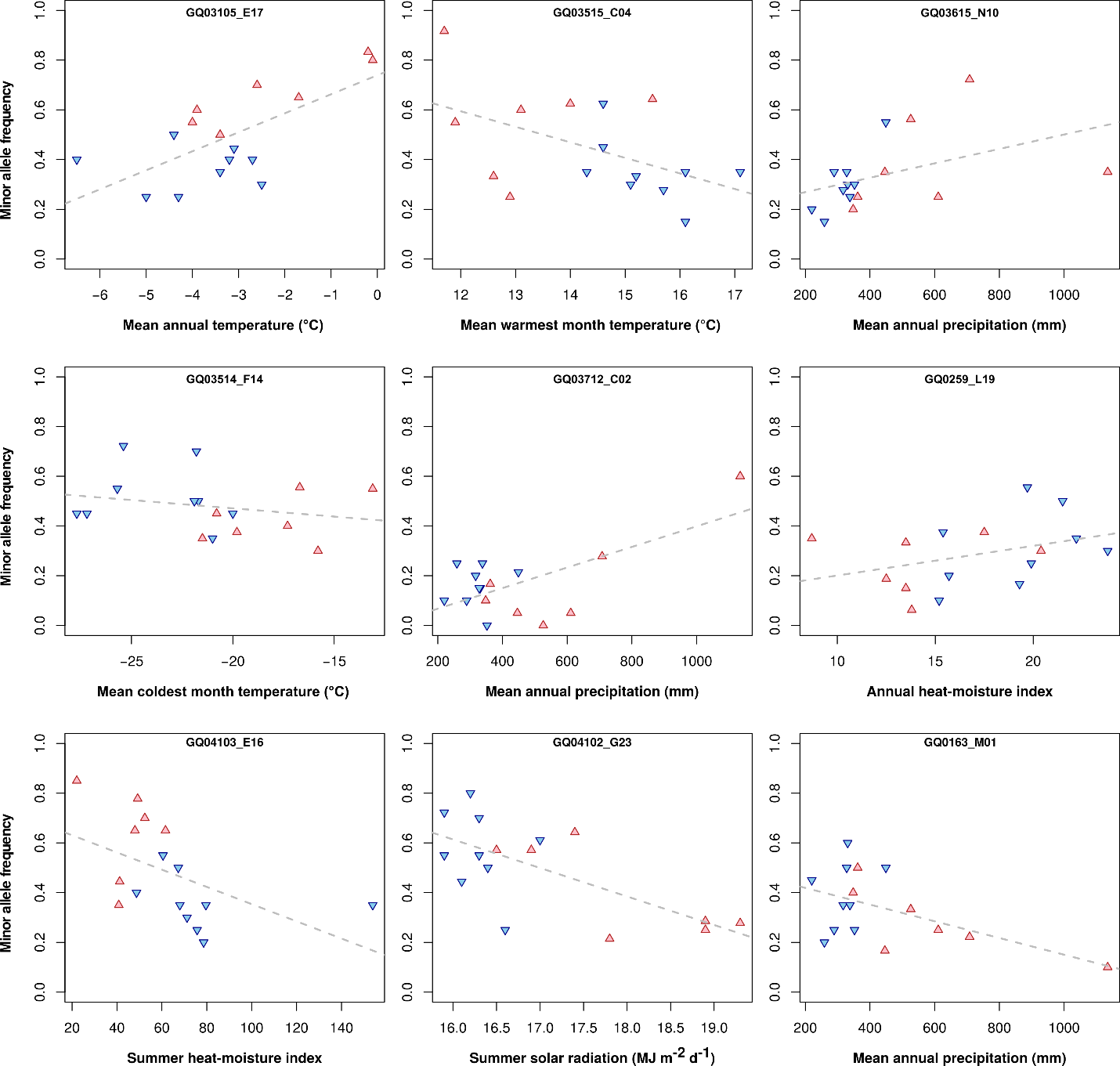


**Fig. S3.** Example of linear relationships found between a climatic variable and allele frequencies at each of the nine candidate genes encompassing the SNP loci jointly identified by the two genotype-environment analyses (pRDA & LFMM). Red and blue triangles represent interior and coastal sampling sites, respectively.


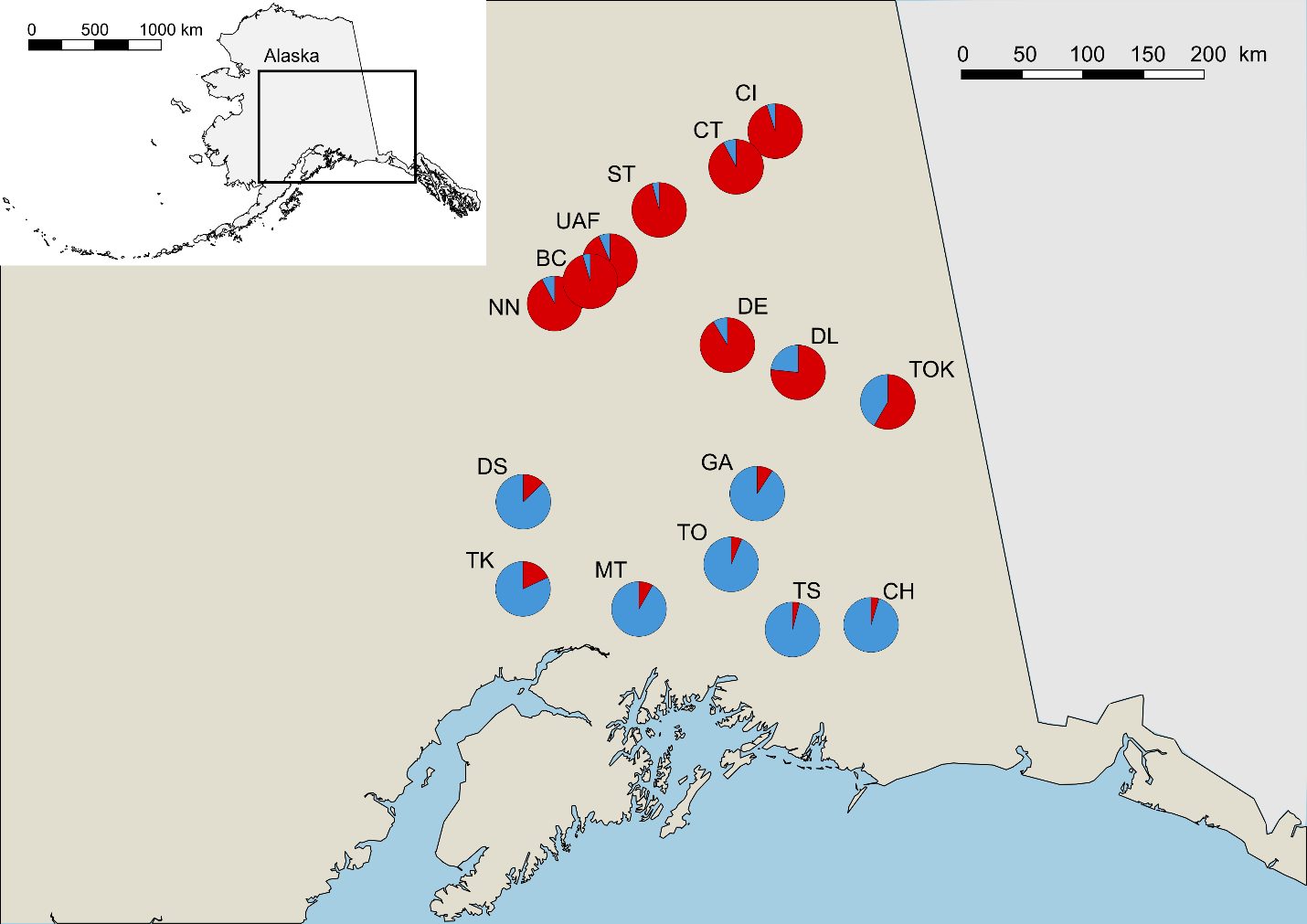


**Fig. S4.** Spatial pattern of genetic structure of sampled black spruce populations. Two distinct groups (*K* = 2) are distributed in spatially distinct clusters.
